# Clustering patients on the basis of their individual course of low back pain over a six month period

**DOI:** 10.1186/1471-2474-12-99

**Published:** 2011-05-17

**Authors:** Iben Axén, Lennart Bodin, Gunnar Bergström, Laszlo Halasz, Fredrik Lange, Peter W Lövgren, Annika Rosenbaum, Charlotte Leboeuf-Yde, Irene Jensen

**Affiliations:** 1 The Karolinska Institutet, Institute of Environmental Medicine, Unit of Intervention and Implementation Research, Sweden; 2 Private practise in Lund, Sweden; 3 Private practise in Stockholm, Sweden; 4 Private practise in Linköping, Sweden; 5 Spine Centre of Southern Denmark, Hospital Lillebælt, Institute of Regional Health Research, University of Southern Denmark, Denmark

## Abstract

**Background:**

Several researchers have searched for subgroups in the heterogeneous population of patients with non-specific low back pain (LBP). To date, subgroups have been identified based on psychological profiles and the variation of pain.

**Methods:**

This multicentre prospective observational study explored the 6- month clinical course with measurements of bothersomeness that were collected from weekly text messages that were sent by 176 patients with LBP. A hierarchical cluster analysis, Ward's method, was used to cluster patients according to the development of their pain.

**Results:**

Four clusters with distinctly different clinical courses were described and further validated against clinical baseline variables and outcomes. Cluster 1, a "stable" cluster, where the course was relatively unchanged over time, contained young patients with good self- rated health. Cluster 2, a group of "fast improvers" who were very bothered initially but rapidly improved, consisted of patients who rated their health as relatively poor but experienced the fewest number of days with bothersome pain of all the clusters. Cluster 3 was the "typical patient" group, with medium bothersomeness at baseline and an average improvement over the first 4-5 weeks. Finally, cluster 4 contained the "slow improvers", a group of patients who improved over 12 weeks. This group contained older individuals who had more LBP the previous year and who also experienced most days with bothersome pain of all the clusters.

**Conclusions:**

It is possible to define clinically meaningful clusters of patients based on their individual course of LBP over time. Future research should aim to reproduce these clusters in different populations, add further clinical variables to distinguish the clusters and test different treatment strategies for them.

## Background

Diagnosis of non-specific low back pain (LBP) is unusual compared to other medical diagnoses as it is based on a set of symptoms rather than a specific pathology. In clinical and epidemiological studies, non-specific LBP is considered to be a single entity even though patients with this diagnosis probably constitute a heterogeneous group. In other words, the term implies that patients with LBP are more or less identical whereas clinicians consider each patient with LBP to be more or less unique.

Research into effective treatments for non-specific LBP probably reflects this heterogeneity since results are conflicting [1,2] and the effect sizes in general small. Identification of the underlying subgroups among these patients would make it possible to target treatments and would therefore potentially influence the outcomes of pain, disability, sick-leave and associated costs.

One approach in this work is to identify prognostic categories of patients. Typically, baseline characteristics have been used to divide patients into prognostic groups. Psychological factors in patients with neck and back pain have shown predictive value for long term sickness absence [3]. Further, clinical development has been used as a prognostic characteristic, and early improvement has been shown to predict improvement in the long-term [4]. However, the topic needs illuminating from all possible angles.

It seems reasonable to assume that patients that react differently to treatment represent different pathologies or psychological profiles, and that patients experience different courses over time both in relation to pain and disability. To date, this is not well understood. One reason is that it has been difficult to follow large groups of patients with frequently repeated assessments over an extended period of time. Therefore, change in the condition is usually presented as the difference in mean values between the start and follow- up measurements. However, this approach ignores both individual variation and fluctuation of the condition over time.

With the help of mobile phones and text messages, it is now possible to collect data frequently and efficiently and to do so over time [5,6]. This method makes it possible to take individual variation into account. Thus heterogeneity in the clinical course can be explored and, perhaps, used to detect underlying patterns. This may be an approach to better understand the diversity of non-specific LBP [7]. In addition, it would be possible to investigate whether such different course patterns are linked to specific background factors and clinical findings [8].

The primary objective of this study was to investigate whether specific clusters can be identified among patients with non-specific LBP, based on an explorative cluster analysis on patients' clinical course pattern of LBP over a 6 month period. Additional objectives were to investigate whether such clusters could be validated against some simple clinical variables, i.e. whether the identified clusters may be clinically relevant.

## Methods

### Patients

To recruit a large number of patients with LBP, a chiropractic patient sample was chosen, as this is the condition most commonly treated by chiropractors in Sweden [9]. The 244 patients included in the study had non-specific LBP, with or without sciatica and no other obvious diseases that could account for the LBP [6]. No categorization according to the duration of their present LBP was used. The patients were of working ages, usually between 18 and 65 years. They had not been under chiropractic care for the past three months. The external validity of this sample has been found to be acceptable, i.e. the general heath and development of pain over time of the sample was compared to that of relevant populations [6]. Patients were not included from the study if pregnant, if unable to understand Swedish, if they did not have a mobile phone, or if they did not know how to use the text message function. Only patients who answered their weekly text messages over 80% (n = 176) of the time were included in the cluster approach. Data collection took place between May 2008 and June 2009.

### Chiropractors

A convenience sample of 35 chiropractors was recruited in a previous study as described elsewhere [6]. In short, they were all members of the Swedish Chiropractic Association (in Swedish: Legitimerade Kiropraktorers Riksorganisation, LKR) to ensure sufficient academic standards of care, and were found to be representative of the LKR as a whole.

### Treatment

The patients were included at the second visit of the current LBP episode to ensure that only those with non-specific LBP were included, assuming that patients with other diagnoses would have been referred to other types of care after the first visit. Therefore, it is also assumed that patients returning for a second visit were treated, and the ensuing course that was studied is thus a clinical course. No restrictions were placed upon the chiropractors in terms of treatment. Previous studies of Swedish chiropractors indicate that most patients would receive spinal manipulation, either directly or mechanically assisted, mobilization, soft tissue treatment, advice, and/or exercises [4,9].

### Ethical considerations

Participation was voluntary. Patients and chiropractors received information about the study and signed informed consent forms. Permission to conduct this study was granted by the local ethics committee 2007/1458-31/4.

### Data

A full description of the data collecting process is reported elsewhere [6]. In short, the participating patients were informed about the study verbally and in writing. If they accepted participation, they filled in a base-line questionnaire with items found to be valid on the study inclusion visit (i.e. the second clinic visit) and signed a consent form. Questions included details of their pain (pain drawing [10] and pain intensity (using a numeric 11-point scale (NRS), anchors at no pain and worst imaginable) [11]), self-reported sick leave (number of days the previous year) [12]) and health ("How would you rate your health? Excellent (1), very good (2), good (3), fair (4), poor (5)"[13] and EuroQol 5, EQ5D [14], weighted score ranging from 0 (death) to 1 (perfect health)). The chiropractor collected information on patients' gender, age and occupation, as well as area of pain, intensity, duration and frequency of pain the previous year, self-reported sick-leave previous year (Y/N), access to a mobile phone and know-how in terms of receiving and sending text messages. During the 4^th ^visit, the patients answered a question regarding improvement [15] (*"How would you rate your improvement (compared to the time of the first treatment)?"*. Answers were provided as a descriptive 5-point scale, ranging from definitely improved to definitely worse).

The questionnaires were sent to the research centre and the respondents were entered into the text message system. The first text message question was sent to the patient the following Sunday afternoon (i.e. shortly after the second visit to the chiropractor) and every Sunday after that for six months. The question was: *"How many days during the previous week was your low back pain bothersome, (i.e. affected your daily activities or routines)? Please answer with a number between 0 and 7*" [16]. In a Danish study on chiropractic patients in which text messages were used to collect data, the weekly number of painful days was found to be closely related to the weekly intensity of the LBP [17]. Therefore, in our study, bothersomeness was used as a measure of the effects of pain on daily life, but was also regarded as a proxy for pain intensity, as it is important to limit the number of questions in text message surveys. Respondents who failed to answer for three weeks in a row were called and kindly reminded to answer. If the respondent could not be reached by phone, a letter was sent to the patient with a reminder to continue answering the text messages.

### About SMS Track^®^

SMS Track^® ^[18], a web-based software program designed for research, was used in this study for the text message questions. The technology enables data from a large number of respondents to be gathered at frequent intervals, in this case once a week. In addition, the data are accessible to the researcher in real time, i.e. data from the text message question can be viewed instantly for each individual and on group level. It is therefore also possible to instantly discover non-responders and discrepant answers, making it possible to contact the patient and provide relevant information. The system has been shown to yield high compliance rates that are unaffected by age, gender and season and it was very user friendly for this study sample [6,19].

### Analysis

The selected analytic approach is person- oriented [20]. This means that the analysis concerns the development of the individual, regardless baseline variables. We hypothesized that the pain course over time would be similar in groups of individuals, and different from the course of other groups. This is different from the more traditional variable-based analysis, in which the hypothesis concerns the association of a baseline variable with the outcome.

The text message replies provided individual curves for each respondent based on the weekly measurement of number of days with bothersome LBP reported for 26 weeks. Visual inspection of the individual curves and of the aggregated curve for all respondents was the starting point of the exploratory cluster analysis.

In cluster analysis, it is practically impossible to use 26 parameters (the weekly data) on which to cluster. Therefore, the courses were condensed as follows: For each individual, two linear regression lines were calculated, which defined first the early and then the later trends over time, the dependent variable being the number of days, and the independent variable the week. An intersection between the two regression lines was calculated. The regression analysis was done using a spline (nonlinear regression) technique which simultaneously estimated the lines and their intersection. Thus, for each patient, the weekly measures were condensed into 4 parameters describing the clinical course. These were: 1) the slope of the regression line describing the early course, 2) the intercept of the regression line describing the early course, 3) the difference in slope between the two regression lines (to describe the change from early to late course) and 4) the intersection estimate (to describe when the change in improvement occurs), the so-called "knot". The curve estimates for each patient were checked for their goodness-of-fit through analysis of their residual variance and R square. It should be noted that the regression lines were simply used to describe the development over time, and that no regression analysis was performed. As the individual curves were described with regression lines, it was necessary to obtain good approximations of the actual courses. Therefore, in order to secure solid curve estimates, only patients responding more than 80% of the time (i.e. 21 weeks or more) were used in the cluster analysis. As the analysis was based on curve estimates, a few missing values did not affect the overall description of the curve in the individuals left for analysis (i.e. those with more than 80% weekly answers).

The four mathematical parameters described above were used in a hierarchical cluster analysis, Ward's method, to detect clusters [21,22]. The parameters were first standardized to counteract differences in scale. In order to determine the optimal number of clusters, a graphical representation specific to cluster analysis, the dendrogram, together with a criterion based on comparisons of the variation within the clusters in relation to the variation between the clusters, the Calinski-Harabasz criterion, was used [22]. Further, using the results of the Ward algorithm as a starting point, a K-means cluster analysis was used to optimize cluster allocations and, if necessary, to reallocate the subjects to other clusters. Reallocations were also evaluated with the Calinski-Harabasz criterion.

The final clusters were then described in relation to initial level of bothersomeness, rate of early improvement and the point of change, making it possible to visualize the course pattern of each cluster.

An attempt was thereafter made to match the clusters with clinical information to ascertain if the clusters were clinically different from each other in other ways as well. A number of clinical variables, namely age, gender, pain intensity, the presence of leg pain, duration of LBP the previous year and self- rated health, as well as two variables of outcome, improvement at the 4^th ^visit and the total number of days with bothersome pain over 26 weeks, were used to describe the clusters. These variables were tested for differences between the clusters with ANOVAs and X^2 ^tests. The clinical variables, the outcome variables excluded, were used in a discriminant analysis (kth-nearest-neighbour) [23] for a multivariate evaluation of cluster differences. Thus, the mathematically obtained clusters were validated by investigating differences in various clinical variables to answer the question: Are the clusters clinically relevant? Analyses were performed using SPSS 17 [24], STATA 10 [25] and Sleipner [26].

## Results

Preliminary visual inspections of the aggregated curves clearly showed that most patients experienced improvement at the beginning of the treatment process. Previous studies have shown that early improvement influences prognosis [15,27,28]. It was also clear from visual inspection that improvement changes or ceases at some point. This change in the clinical course might simply indicate a slower speed of recovery, but it might also indicate the start of a relapse.

### The study sample

In total, 262 patients were recruited in the study, but 18 patients (< 7%) dropped out. Sixty eight patients did not satisfy the criterion of answering more than 80% of the time, while 176 patients (72% of the possible sample) did. A further 11 patients were removed from the cluster analysis as described below. Thus 165 patients were left in the final cluster solution (262-18-68-11 = 165).

Baseline data on age, gender, pain intensity, presence of leg pain, duration of previous pain and self- rated health, as well as the outcomes improvement at the 4^th ^visit, which took place within 14 days for 69% of the sample, and the total number of days with bothersome LBP during the study period are presented for the patients included (n = 165) and excluded (n = 68) from the analysis in Table 1. The baseline characteristics of the patients included and excluded from the cluster analysis were different only in the matter of improvement by the 4^th ^visit, as significantly fewer patients among the excluded patients regarded themselves as "definitely improved".

**Table 1:** Baseline variables and major outcomes of patients in the study and across clusters

	Patients in the study n = 165	Patients excluded n = 68	Cluster 1, n = 43	Cluster 2, n = 23	Cluster 3, n = 72	Cluster 4, n = 27
Age, mean, (range) *	45 (21-69)	42 (16-64)	42 (21-62)	46 (33-63)	45 (24-69)	49 (27-67)
Gender, % male	54	50	56	46	49	70
Pain, VAS mean, (SD)*	4.4 (2.1)	4.4 (2.4)	3.7 (2.6)	5.3 (1.9)	4.6 (1.9)	4.2 (2.1)
Leg pain, %	48	48	35	47	53	57
> 30 days of pain previous year, % *	58	61	56	30	64	70
EQ5D baseline, mean (range)	0.73 (-0.05-1.00)	0.78 (0.70-0.84)	0.79 (-0.05-1.00)	0.69 (0.26-0.84)	0.72 (-0.05-1.00)	0.70 (-0.05-0.84)
Improvement by 4^th ^visit, %	73	58	80	73	68	74
Total number of days with bothersome pain, mean (SD) *	38 (35)	35 (32)	33 (37)	28 (20)	35 (31)	64 (39)

* Denotes statistically significant differences between clusters.

As regression lines were used to define the individual profiles, patients (n = 4) with a constant response (i.e. course was a horizontal line) could not be described with this method. Two of these individuals reported zero days with bothersome LBP throughout the period and 2 reported 7 days throughout the period, making it impossible to consider these patients a cluster on their own. Their data were excluded from the analyses as they were also found to be a heterogeneous group also in relation to several baseline characteristics.

A further 7 patients' curves defied the initial step of the hierarchical cluster analysis by not matching any other respondent in the very first step of the analysis. They were isolated outliers and did not appropriately fit any cluster. Technically, these cases are considered a separate heterogeneous group, and should therefore be excluded from further cluster analyses. Descriptive analysis of these 11 patients' baseline variables revealed that most were female, that they were more likely to have had leg pain and that they had more days with bothersome pain than the rest of the study group (data not shown).

### Clusters based on the course of low back pain

Ward's method with the applied Calinski-Harabasz criterion suggested 4 definite clusters, which were largely confirmed by the K-means method since only 15% of respondents were reallocated. Scrutiny of the differences between Ward's and the K- means methods revealed that one cluster (Cluster 2 described below) was intact in this process. Further, in another cluster (Cluster 3 described below) only one individual was moved. The two remaining clusters contained a "core" of individuals who resembled each other and who were therefore not reallocated. However, there were a few individuals who were more difficult to allocate according to the cluster parameters, and they were therefore moved between these clusters in the reallocation process.

The final 4 clusters from the K-means method, ranging in size from 23 to 72 individuals, are described in terms of their spline regression parameters in Table 2.

**Table 2:** Spline regression parameters of the final 4 clusters

	Cluster 1, n = 43, mean	Cluster 2, n = 23, mean	Cluster 3, n = 72, mean	Cluster 4, n = 27, mean
Intercept, First regression	1.45	9.44	5.76	5.07
Slope, First regression	0.06	-3.08	-1.06	-0.26
Slope, Second regression^1^	-0.02	-0.03	-0.02	-0.09
Intersection between first and second regression, "knot"^2^	4.84	2.77	4.40	12.04

^1 ^The parameter used for the cluster analysis was actually the difference in slopes between the first and second regressions. To facilitate the interpretation, we show the parameter here as the slope of the second regression.

^2 ^This parameter is scaled in weeks.

The second best fit of the data using the Calinski-Harabasz criterion was a 7-cluster solution. These clusters ranged in size from 3 to 59 individuals. The most noteworthy difference between the two solutions was that clusters 2 and 4 remained unchanged in the agglomeration process, suggesting stability. The transitions between the 7- cluster and 4- cluster solutions include the merger of two sub clusters into Cluster 1 and the merger of three sub clusters into Cluster 3. For Cluster 1, one small sub cluster of only 3 individuals was merged into an existing sub cluster of 33 individuals, rendering Cluster 1 relatively stable between the 7- cluster and the 4-cluster solutions as well. The merging of clusters from the 7- to the 4- cluster solutions available through the hierarchical cluster technique thus gave valuable insight in the properties of the finally adopted solution.

### Mathematical and characteristic clinical properties of Clusters 1, 2, 3 and 4 (Tables 1 and 2, Figures 1, 2, 3, 4 and 5)

**Figure 1:**
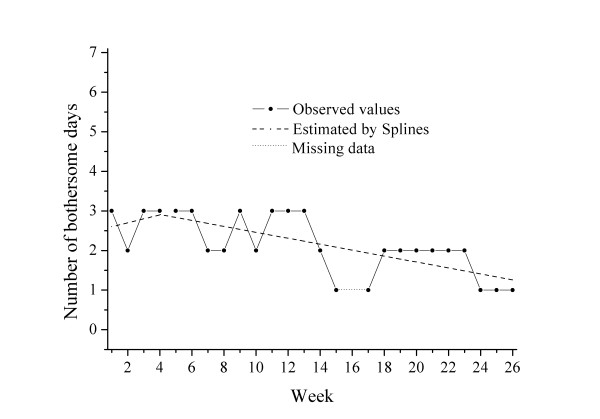
**Example of an individual course in Cluster 1: "stable"**.

**Cluster 1 **(n = 43) was characterized by low bothersomeness initially (intercept 1.5), minor deterioration in the early phase (slope 0.06), a turning point at 4.8 weeks and then a slight improvement in the later phase. Overall, the course of this group of patients was rather stable. One example of the course pattern and the individual regression lines in a patient from this "stable" cluster is shown in Figure 1.

The pain level of this group was rather low (VAS score 3.7 at baseline). The patients were characterized by being the youngest (mean age 41.6 years) and having the best self- estimated general health (EQ5D weighted score 0.788), and 80% reported definite improvement by the 4^th ^visit. The mean total number of bothersome days during the follow-up period for this group was 32.8 days.

**Figure 2:**
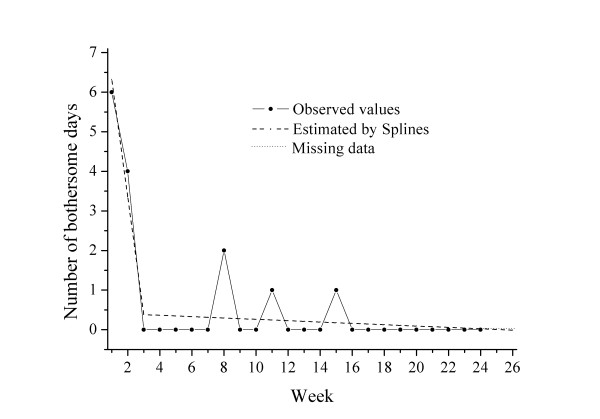
**Example of an individual course in Cluster 2: "fast improvers"**.

**Cluster 2 **(n = 23) was characterized by a high degree of bothersomeness initially (intercept 9.4), rapid improvement in the early phase (slope -3.08), a turning point as early as at 2.8 weeks and slower improvement thereafter. One example of a course pattern and the individual regression lines in a patient from this "fast improvers "cluster is shown in Figure 2.

These patients had the highest pain levels (VAS score of 5.3) and the poorest self- rated health (EQ5D weighted score 0.685) at baseline, were mostly female (64%) and a majority (almost 70%) had experienced pain less than 30 days the previous year. The mean total number of bothersome days for this group was 28.3 days during the follow-up period, the lowest of the 4 clusters.

**Figure 3:**
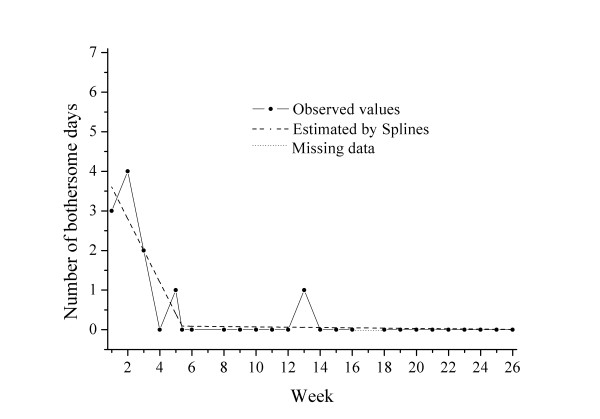
**Example of an individual curve in Cluster 3: "typical patient"**.

**Cluster 3 **(n = 72) was characterised by a fairly high degree of bothersomeness initially (intercept 5.8), fairly rapid improvement (slope -1.06), a turning point at 4.4 weeks and slower improvement thereafter. One example of a course pattern and the individual regression lines in a patient from this "typical" cluster is shown in Figure 3.

Concerning baseline variables, this was the "typical" group; age, gender, pain intensity and duration of previous LBP resembled that of the full cohort, as did the course over time. The mean total number of bothersome days for this group was 34.9 days.

**Figure 4:**
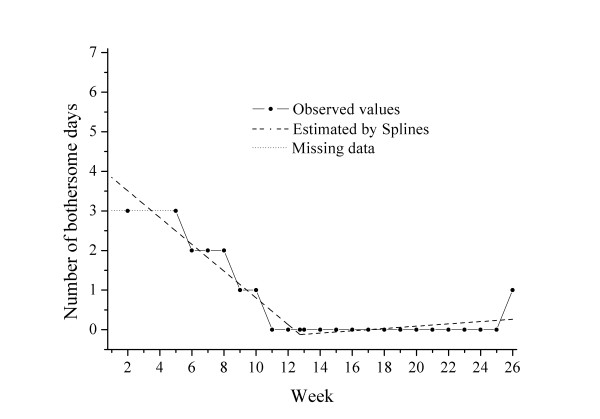
**Example of an individual curve in Cluster 4: "slow improvers"**.

**Cluster 4 **(n = 27) was characterised by medium bothersomeness initially (intercept 5.1), slow improvement (slope -0.26) and a late turning point at 12.0 weeks with continued slow improvement thereafter. One example of a course pattern and the individual regression lines in a patient from this "slow improvers" cluster is shown in Figure 4.

These patients were older (mean age 49.4 years), were mostly men (70%) and a large proportion (70%) of the patients had reported more than 30 days of LBP the previous year. Still, a majority of the patients (74%) rated themselves as "definitely improved" by the fourth visit. The mean total number of bothersome days for this group was 64.3 days, the highest of the 4 clusters.

**Figure 5:**
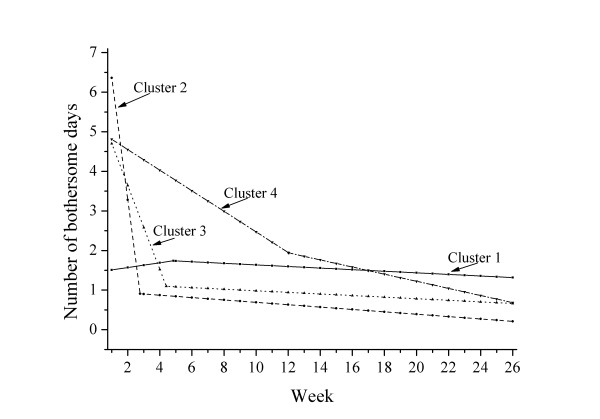
**The four clusters described by aggregated spline regression lines**.

The estimated curves of the different clusters are visualized in Figure 5.

### Statistically significant differences between clusters

Analysis of the differences in cluster baseline characteristics and outcomes is shown in Table 1. Significant differences were found at baseline in age (older in Cluster 4), pain (highest in Cluster 2) and duration the previous year (most patients with LBP with more than 30 days the previous year in Cluster 4) as well as in the outcome of total number of days with bothersome pain (more in Cluster 4).

The multivariate discriminant analysis showed that the error rate in predicting cluster membership based on the clinical variables was 22.4% for the 132 subjects that we were able to classify. The excluded patients were due to missing values for some baseline variables. This error rate is considerably lower than the error rate for a completely random allocation of around 75%.

## Discussion

This study shows that it is possible to define distinct clusters of patients with LBP based on their individual clinical course over time. The analyses of the few available clinical characteristics of the clusters suggest that these clusters may be relevant for future research on prediction, prognosis and ultimately specific treatment regimes. However, more and better clinical, psychological, and social information would be needed for a more detailed analysis.

### Clinical significance

By applying descriptive terms and clinical information to our clusters, we have shown that it is possible to distinguish specific clinically recognisable subpopulations within the group of non-specific LBP. The patients in the study sought care for their LBP, but obviously the conclusion cannot be made that chiropractic care was responsible for the observed clinical course, and the observed course may as well represent the natural course. As the patients in this study had differing duration of LBP, the described cluster could possibly represent different "windows" of a common clinical course, the way it would appear in daily practice.

For the individual clinician, it may be useful to have an idea of the likely clinical course. In general patients improved, but the rate of improvement was more rapid for some (Cluster 2) and slower for others (Cluster 4). In clinical practise, this means that most patients should be improved by the 4^th ^or 5^th ^week, which should be the time for re-examination and reflection regarding patient outcome. In a more detailed analysis, young and relatively healthy patients with low levels of bothersomeness and pain (Cluster 1) can be expected to experience a relatively stable course over time and very bothered patients (Cluster 2) can be expected to improve rapidly, whereas those in the older age groups with longstanding pain (more than 30 days the previous year) (Cluster 4) can be expected to respond more slowly. In the clinical setting, patients who are relatively unchanged (Cluster 1) or who improve very slowly (Cluster 4) may be difficult to manage. Luckily, the majority of these patients regard themselves as "definitely improved" at the 4^th ^visit. This should make clinical management easier, as treatment may otherwise be considered disappointing to patients who are experiencing a stable course or slow recovery. Thus, knowledge about likely course is important for patient education.

It is perhaps strange that the patients in Cluster 1 regard themselves as" definitely improved" as they, in fact, have a relatively stable course. It is possible that these patients already had improved considerably when included in the study. It is also possible that their pain is not bothersome to them, so that our chosen outcome measure fails to be responsive in this respect.

### Previous research

The overall picture of the patient population is in concordance with the results from other research on patients with non-specific LBP [29], on a group level improvement is noted after consultation. The rate of improvement is different between the clusters, as defined by the slopes and turning points. The "cut-point", where the rapid improvement of the first few weeks becomes slower, is in concordance with a previous study for predicting outcome [15]. Most of the patients in this study regard themselves as "definitely improved" by the fourth visit, which is similar to a pilot study of 158 patients with LBP who were treated in chiropractic practice [30].

In a study on *prognostic factors *for acute LBP, poor self- rated health was found to predict poor recovery [31] in patients who received medication as the only treatment, while in our study the cluster with the poorest self- rated health had the quickest recovery rate. Thus, compared to previous studies, this seems like an illogical finding. It is possible that highly bothersome LBP influenced the attitudes towards general health in this group of patients.

In a previous Swedish study on chiropractic patients [32], those experiencing LBP for *more than 30 days *the previous year had a poorer outcome in both short- and long- term. In our Cluster 4, in which 70% of patients reported LBP for more than 30 days the previous year, the rate of improvement was the slowest. In a study on LBP in an occupational setting, patients with a previous duration of more than 30 days also had a poorer chance of long-term recovery [33], using zero days of pain as a measure of recovery. In our cohort, however, it was rare for patients to report zero days of bothersome pain for any length of time.

### Previously described subgroups based on clinical course (Table 3)

To our knowledge, two previous attempts have been made to subgroup patients with LBP using cluster analysis [34,35]. In an English cross-sectional study by Langworthy et al. [34], baseline variables on the details of pain obtained from patient files were the starting point for the hierarchical cluster analysis, which revealed 2 main subgroups that were differentiated by constant and fluctuating pain, respectively. These results cannot readily be compared to our results, as our approach is based on the clinical course of LBP, which is assumed to be fluctuating. Further, our patients were assessed prospectively over 6 months.

In a longitudinal study by Chen et al, patients sick-listed for LBP were interviewed at baseline and at 3-5 points over the following year concerning pain intensity [35], and then a K-means cluster analysis was performed. Five clusters were identified based on changes in pain intensity over time. This study is similar to ours in the longitudinal assessment, but differs with respect to the population and number of measurements. Some of the clusters found seem similar to the clusters identified in our study.

A different approach, latent class analysis, was used by a Swiss research group, Tamcan et al., to subgroup patients on the basis of the natural course of LBP [36]. The pain data from weekly diaries over one year suggest that four major subgroups exist: 1) severe persistent, 2) moderate persistent, 3) mild persistent and 4) fluctuating. However, the value of diary data has been questioned as people very often backfill such records, rendering them prone to recall bias and memory decay [37].

Using the same frequent data collection technology as in our study, researchers in Denmark, Kongsted et al., have previously used a purely visual approach to describe patients' course patterns 1) within the first 4 weeks and 2) between weeks 5 and 18. They defined, pre-hoc, 13 possible categories and subsequently found 9 of these [5]. Despite the difference in study duration between the Danish study and ours, it is possible to find similarities in some of the results regarding course pattern and total number of days with LBP as seen in Table 3. A comparison of sizes between the Danish categories and our clusters also makes sense; the largest cluster in our study is very similar to one of the largest in the Danish study, and the proportional size of the other clusters seems to be comparable.

**Table 3:** Comparing the clusters in this study with subgroups in previous studies on patients with LBP

**Langworthy **[34]	**Chen **[35]	**Tamcan **[36]	**Dunn **[38]	**Kongsted **[5]	This study
Constant pain	Continous high	Severe persistent	Severe chronic		
	
		Moderate persistent			Cluster 4
	
		Mild persistent	Mild persistent	Unchanged, improves thereafter	Clusters 1 & 3

Fluctuating pain	Fluctuating	Fluctuating	Fluctuating	Improved, Fluctuates thereafter	Cluster 3 Cluster 1
	
			Recovering	Improved, remains so	Cluster 2
	
	Large reduction				Cluster 4

In a recent British study by Dunn et al. of patients with back pain in the primary care sector, patients were monitored with monthly questionnaires over 6 months [38]. Four clusters based on pain intensity were described using latent class analysis: 1) mild persistent, 2) recovering, 3) severe chronic and 4) fluctuating. Unlike the clusters revealed in our study, these subgroups could not be described in terms of age or gender, but according to several clinical variables. Moreover, the pain profiles of their clusters were on different "levels", with the recoverers showing the lowest level of pain, and the "severe chronic" patients showing the highest level of pain. It is therefore difficult to perfectly match their subgroups to our clusters (Table 3). This is possibly because all of the patients in our study were measured weekly, and thus even minor fluctuations were captured, whereas measurements in the English study were recorded monthly. This, of course, raises the question regarding the optimal interval for measuring a fluctuating condition such as LBP. Is it adequate to measure monthly, and does the detail provided by weekly measurements merely add "noise" in terms of capturing important course patterns? In other words: are the clusters obtained through weekly measurements a mere reflection of different phases of the LBP condition and if so, are they relevant in the long term? This obviously requires further study.

### Methodology

The use of text messages to collect data opens up possibilities for accurate descriptions of fluctuating conditions since it is possible to obtain detailed information on changes over time. This study has shown that clusters can be defined by describing the clinical course in mathematical terms. The question of clinical meaningfulness is inherent in cluster analysis. Therefore, the examination of the different solutions in the objectively mathematically formed clusters should be supplemented with a subjective clinical evaluation, as is the case in this study.

The patients in this study were included when they consulted for LBP. Therefore, they are expected to have pain at the time of inclusion, and to improve with time. We do not, however, have data on the frequency of treatments, and can therefore not evaluate the influence of treatment on the different clusters. Further, to minimize the burden on the data collecting clinicians, no record of patients excluded or refusing to participate was kept. This is a limitation and could cause bias as the patients who did not register may have had a different pain profile.

Only data from the highly compliant responders were used in the analysis as the course descriptions were the basis for our clustering and solid estimates were needed. This may have introduced bias in our final cluster solution. Possibly more clusters or clusters with a different profile would have been the result if we had included all the patients. However, we regard the cluster solution presented here as solid, as it is tested with two different approaches (Ward's and K means) and criteria for optimal between-cluster distances (Calinski- Harabasz). The multivariate analysis for predicting cluster membership had an error rate which we consider low, further strengthening the solidity of our clusters.

Other alternatives than cluster analysis based on spline regression parameters exist for this kind of data such as latent class analysis. Our choice of method was based on a desire to obtain parameters for the gradients in pain reduction in the early and late phases and an estimate of the shift in pain reduction, i.e. the knot in the spline regression. All these parameters are easily interpreted in the clinical practice.

This study is explorative, i.e. we had no hypothesis regarding the number or size of clusters in our population. The focus of the solution presented here is simplistic clarity, the 4-cluster solution, whereas the 7-cluster solution presents smaller but more homogeneous clusters with detailed clinical information. At this point in time, the clinical importance of the diversity is unknown and would be presented at the expense of generalizability. Larger study samples would be needed to further scrutinize the clinical significance of the 7 cluster solution.

In the context of subgrouping research, this study can be described as a data-driven approach since the clusters were determined on the basis of outcome (the clinical course). This type of explorative cluster analysis can be viewed as hypothesis-setting since hypotheses about underlying clusters were postulated retrospectively [39]. Consequently, the results obtained in this study need to be validated in subsequent studies, in which clusters are tested in a pre hoc fashion.

### Research perspectives

Future studies should aim to reproduce these clusters in different patient populations. To add meaningfulness, more clinical variables should be added in an attempt to characterize each cluster, and include also psychosocial variables. This might improve the descriptive characteristics of the smaller groups found in the 7-cluster solution of this study. Further, different treatment strategies should be assessed for each group separately. If these study strategies prove to be effective, proper allocation of resources would ensure optimal efficacy in the treatment of "non-specific" LBP.

## Conclusion

It was possible to define clusters with a mathematical description of each individual clinical course in this population of patients with LBP. In the preliminary analyses, the defined clusters seem to make clinical sense and concur to some extent with those defined in previous studies.

## Competing interests

The authors declare that they have no competing interests.

## Authors' contributions

IA was responsible for the design of the study and the supervision of data collection, participated in the analysis of data and was responsible for the manuscript preparation. LB was responsible for the data analysis. LH, FL, PWL and AR were involved in the design and the supervision of data collection. GB, CLY and IJ were supervising the study process and were involved in the manuscript preparation. All authors revised and approved the final manuscript.

## Pre-publication history

The pre-publication history for this paper can be accessed here:

http://www.biomedcentral.com/1471-2474/12/99/prepub
